# Specific Gene Expression in *Pseudomonas Putida* U Shows New Alternatives for Cadaverine and Putrescine Catabolism

**DOI:** 10.3390/genes14101897

**Published:** 2023-09-30

**Authors:** Luis Getino, Alejandro Chamizo-Ampudia, José Luis Martín, José María Luengo, Carlos Barreiro, Elías R. Olivera

**Affiliations:** Área de Bioquímica y Biología Molecular, Departamento de Biología Molecular, Universidad de León, 24007 León, Spain; luis.getino@unileon.es (L.G.); alejandro.chamizo@unileon.es (A.C.-A.); jmartf12@estudiantes.unileon.es (J.L.M.); jmluer@unileon.es (J.M.L.); c.barreiro@unileon.es (C.B.)

**Keywords:** *Pseudomonas putida*, amines, polyamines degradation pathways, putrescine, cadaverine, ɣ-aminobutyrate (GABA), δ-aminovalerate (DAVA), ɣ-aminobutyrate aminotransferase (GabT), putrescine-pyruvate aminotransferase (SpuC)

## Abstract

*Pseudomonas putida* strain U can be grown using, as sole carbon sources, the biogenic amines putrescine or cadaverine, as well as their catabolic intermediates, ɣ-aminobutyrate or δ-aminovalerate, respectively. Several paralogs for the genes that encode some of the activities involved in the catabolism of these compounds, such as a putrescine-pyruvate aminotransferase (*spuC1* and *spuC2* genes) and a ɣ-aminobutyrate aminotransferase (*gabT1* and *gabT2* genes) have been identified in this bacterium. When the expression pattern of these genes is analyzed by qPCR, it is drastically conditioned by supplying the carbon sources. Thus, *spuC1* is upregulated by putrescine, whereas *spuC2* seems to be exclusively induced by cadaverine. However, *gabT1* increases its expression in response to different polyamines or aminated catabolic derivatives from them (i.e., ɣ-aminobutyrate or δ-aminovalerate), although *gabT2* does not change its expression level concerning no-amine unrelated carbon sources (citrate). These results reveal differences between the mechanisms proposed for polyamine catabolism in *P. aeruginosa* and *Escherichia coli* concerning *P. putida* strain U, as well as allow a deeper understanding of the enzymatic systems used by this last strain during polyamine metabolism.

## 1. Introduction

In 2017, the World Health Organization (WHO) estimated that there were 600 million cases of illness caused by unsafe food, with the death of 420,000 people each year. Food quality is an important factor that must be considered to safeguard the health of consumers [1]. Foodborne illnesses can be caused by bacteria, viruses, parasites, contaminants, or allergens, among others. Although these organisms are the main causes of food poisoning, the presence of molecules of biological origin, such as mycotoxins, aflatoxins, or biogenic amines, is of great importance as a marker of food safety [2]. Thus, due to their toxigenic potential, the presence and concentration of biogenic amines and polyamines in food require strict control [2]. These amines can be found in foods that have undergone biological processing, being present in fermented beverages such as wine and beer, dairy products (cheese, yogurt, or fermented milk), fresh fish and fishery products, or meat and meat products [3,4,5,6,7]. Moreover, the food occurrence of these compounds can be related to improper handling and storage [8].

On the other hand, polyamines are present in physiologically appropriate concentrations in almost all organisms, where they are involved in a variety of cellular processes, such as gene regulation, survival, stress response, fulfilling biochemical roles in the synthesis, maintenance, and stability of nucleic acids and proteins [9,10,11]. However, intracellular polyamine concentrations show variations between cells and organisms. Thus, depending on the activation state of murine macrophages, putrescine, spermidine, and spermine have been documented to range from 250 to 1750 pmol determined in 5 × 10^6^ macrophages. On the other hand, concentrations of putrescine in *E. coli* have been documented in a range from 10 to 30 mM, although in other bacteria, the determined content ranged between 0.1 and 0.2 mM [12]. Moreover, polyamines seem to be very ubiquitous molecules that have also been found in humus in nM concentrations per gram of sample, with an ecotoxicological effect, because an increase in these concentrations could trigger variations on the microbiota from these soils due to toxic effects over some of the bacteria present [13]. 

It has been well documented that an exogenous consumption of these compounds from spoiled food could promote adverse effects on human health, triggering deleterious effects and causing specific symptoms. Moreover, the concentration of polyamines exceeding physiological levels in the human body has been reported to be linked to tumoral, inflammatory, and apoptotic events and also to specific infections, mainly by intracellular pathogens [14,15,16].

**Figure 1:**
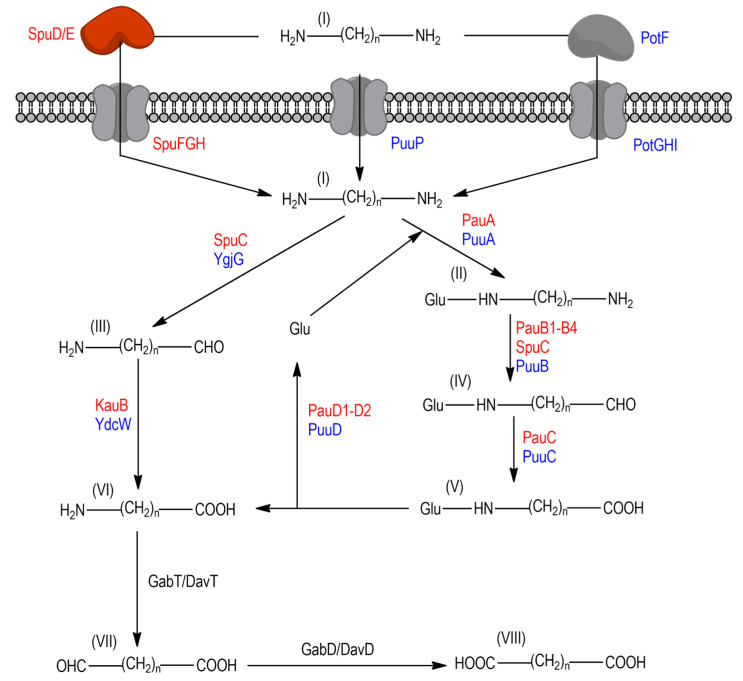
Direct and γ-glutamylation pathway for putrescine and cadaverine catabolism. *E. coli* proteins are marked in blue, *P. aeruginosa* proteins in red, and those homolog proteins present in both bacteria are written in black color. Enzymes: SpuD (putrescine-binding periplasmic protein); SpuE (spermidine-binding periplasmic protein); SpuF/PotG (spermidine/putrescine import ATP-binding protein); SpuG/PotH (polyamine transport protein); SpuH/PotI (polyamine transport protein); PuuP (putrescine importer); SpuC (putrescine:pyruvate aminotransferase); YgjG (putrescine aminotransferase); KauB (4-guanidinobutyraldehyde dehydrogenase); YdcW (γ-aminobutyraldehyde dehydrogenase); PauA (glutamine synthetase); PauB (g-glutamyl-polyamine oxidoreductase); PauC (aldehyde dehydrogenase); PauD (glutamine amidotransferase); PuuA (γ-glutamylputrescine synthetase); PuuB (γ-glutamyl-polyamine oxidoreductase); PuuC (NADP/NAD-dependent aldehyde dehydrogenase); PuuD (γ-glutamyl-γ-aminobutyrate hydrolase); GabT/DavT (γ-aminobutyrate aminotransferase/δ-aminovalerate aminotransferase); GabD/DavD (succinate-semialdehyde dehydrogenase [NADP(+)]/glutarate-semialdehyde dehydrogenase). Glu (Glutamate); when *n* = 4 (**I**) putrescine, (**II**) γ-glutamylputrescine (**III)** γ aminobutyraldehyde, (**IV**) γ glutamyl aminobutyraldehyde; (**V**) γ-glutamylaminobutyric acid; (**VI**) γ-aminobutyric acid; (**VII**) succinic semialdehyde; (**VIII**) succinic acid; whereas *n* = 5 (**I**) cadaverine; (II) δ-glutamylcadaverine; (**III**) δ-aminovaleraldehyde; (**IV**) δ-glutamylaminovaleraldehyde; (**V**) δ-glutamylaminovaleric acid; (**VI**) δ-aminovaleric; (**VII**) glutarate semialdehyde (**VIII**) glutarate.

In addition to their endogenous production fulfilling physiological functions, some bacteria can use polyamines as a carbon and/or nitrogen source through different pathways to support growth and functionality, as has been documented in model organisms such as *Escherichia coli*, *P. aeruginosa,* and *Streptomyces coelicolor* [17,18]. On these pathways, catabolism of putrescine and cadaverine always leads to the production of ɣ-aminobutyrate (GABA) and δ-aminovalerate (DAVA) as metabolic intermediates, respectively (Figure 1). Interestingly, it has also been documented that putrescine at low concentrations (0.15 mM) could be toxic for *Anacystis nidulans* [19]. In *E. coli* K12, two proteins have been described: a YgjG (putrescine aminotransferase) able to deaminate putrescine, cadaverine, and, with lower efficiency, spermidine [20], and a YdcW, a ɣ-aminobutyraldehyde dehydrogenase which oxidates the aldehyde generated after deamination to render a nonproteinogenic amino acid (GABA or DAVA) that will be later integrated into the general metabolism of the microorganism using specific enzymatic functions [21].

Likewise, an alternative route for polyamine catabolism has also been described in *E. coli* K12, which involves the generation of ɣ-glutamyl intermediates [22]. This process starts through the use of an ATP-dependent ɣ-glutamyl putrescine synthetase (PuuA), linking a glutamate molecule to one of the amino groups from putrescine. Subsequently, PuuB (ɣ-glutamyl putrescine oxidoreductase) eliminates the free amino group, producing an aldehyde derivative. This aldehyde, through the action of a NAD(P)-dependent aldehyde dehydrogenase (PuuC) and a ɣ-glutamyl-ɣ-aminobutyrate hydrolase (PuuD), results in the final product GABA, with the concomitant recycling of glutamate [22]. However, this metabolic pathway has not been fully described, although it has been observed that PuuA can recognize cadaverine as a substrate, undoubtedly linking the *puu* genes with cadaverine catabolism [23]. Notably, studies with *E. coli* K12 strains affected in the *puuA*, *puuB*, and *puuC* genes, as well as the *ygjG* and *ydcW* genes, reveal the existence of two independent sets of enzymes capable of completely biotransforming putrescine into succinate [24]. Studies on GABA degradation have reported the existence of several paralogs of *gabT* and *gabD*, with at least one copy of *gabT* being inducible by polyamines and catabolic intermediates and another whose expression is not affected by putrescine, cadaverine, and their intermediates [25].

Similarly, in *P. aeruginosa* PAO1, the existence of analogous pathways to those found in *E. coli* has been suggested. Regarding the deamination of putrescine or ɣ-glutamyl putrescine, both reactions are catalyzed by SpuC (putrescine-pyruvate aminotransferase), which utilizes pyruvate as a substrate, generating alanine [17,26]. However, it has been shown that other genes participate at this point, such as *pauB*, indicating the complexity of the catabolic pathways in *P. aeruginosa* PAO1 [27,28]. Four genes encoding γ-glutamyl-polyamine deamination enzymes capable of releasing free ammonium from polyamines have been identified. Studies in mutants lacking the *spuC* and the *pauB1-4,* although expressing different *pauB* genes, show the ability of this strain to grow using putrescine as the sole carbon source. Thus, strain derivatives expressing *pauB1*, *pauB3*, or *spuC* were able to grow using putrescine, whereas the growth on cadaverine was recovered by the expression of *pauB1* or *spuC* [29].

Finally, Chou et al. demonstrated, through transcriptome analysis, the presence of ɣ-aminobutyrate aminotransferases (*gabT*), whose expression is linked to the presence of putrescine in the medium [27].

These data strongly suggest that there are different proteins involved in the GABA and DAVA catabolism in both *Pseudomonas* and *Escherichia*. Also, they reflect a complex mechanism of differential expression of all the genes involved.

Moreover, it has to be considered that some *E. coli* and *P. aeruginosa* strains are pathogens for human beings. Considering that, it could be feasible that polyamine catabolism in these bacteria could show us new therapeutic targets for the treatment of these infections. Taking this in mind, any contribution to mechanistic issues and alternatives for the degradation of polyamines by microorganisms should be welcome.

*P. putida* strain U has been used as the main model to define biogenic amine degradation (phenylethylamine, tyramine, dopamine, histamine, and others) [30,31,32]. Thus, considering the deleterious effects that an overdose of polyamines from unsafe food could have on human beings, as well as the potential use of the microbial catabolic pathways for these compounds as targets for the treatment of infections, we aimed to study the degradation of these kinds of compounds in this model bacteria. Although this bacterium could not be included in the food chain and has not been related to infective processes, it provides interesting catabolic alternatives that could contribute to a better understanding of polyamine metabolism. Thus, in this study, we describe the existence of different paralog genes coding putrescine-pyruvate aminotransferase and ɣ-aminobutyrate aminotransferase activities. Moreover, we analyze the differential expression of these genes in response to the presence of cadaverine, putrescine, or catabolic derivatives from them (γ-aminobutyrate and δ-aminovalerate), which allows us to suggest that cadaverine and putrescine metabolism in *P. putida* U could show an increase in complexity, at least in some of the steps of the catabolic routes.

## 2. Materials and Methods

### 2.1. Biochemical Reagents

Biochemical reagents (media, antibiotics, and carbon sources) were purchased from Condalab (Madrid, Spain), Sigma-Aldrich (San Luis, MO, USA), Alfa-Aesar (Haverhill, MA, USA), and Acros Organic (Geel, Belgium). Molecular biology products were supplied by Thermo Scientific (Waltham, MA, USA) and Quiagen (Hilden, Germany). Other products used were supplied by Thermo Scientific (Waltham, MA, USA) and VWR (Radnor, PA, USA) with analytical quality or high-performance liquid chromatography (HPLC) grades. Macrogen (Seoul, Pepublic of Korea) provided the oligonucleotides. Molecular biology reagents were supplied by Promega (Madison, WI, USA) and Applied Biosystem (Waltham, MA, USA).

### 2.2. Microorganisms and Culture Conditions

The main strain used in this study is *Pseudomonas putida* U (Colección Española de Cultivos Tipo, CECT4848), a Gram-negative bacterium that exhibits resistance to rifampicin (25 µg/mL), ampicillin (100 µg/mL), and chloramphenicol (30 µg/mL).

For routine maintenance, the strain was grown at 30 °C on LB plates containing 2% (w/v) agar and supplemented with rifampicin [33]. For liquid cultures, *Pseudomonas* was previously grown in minimal medium (MM) plates supplemented with 10 mM citrate and rifampicin and containing 2% (*w*/*v*) agar [34].

To initiate experiments, an inoculum was prepared in the liquid minimal medium under the same conditions as the solid culture and incubated with agitation at 250 rpm and 30 °C along the needed time for each experiment. The growth kinetics and gene expression analysis were carried out using cultures made in 500 mL flasks containing 100 mL of minimal medium supplemented with the carbon sources of interest (citrate, putrescine, GABA, cadaverine, or DAVA) at a concentration of 10 mM, 250 rpm shaking, and 30 °C temperature in the incubator. Selected strains for each experiment were seeded at an optical density (OD) of 0.05 measured at 540 nm, and the growth was determined periodically as an increase in the absorbance at 540 nm. In some experiments, cultures in minimal media containing the corresponding carbon sources at a concentration of 10 mM were supplied with 1mM of cadaverine or putrescine, used as a potential inducer of specific metabolism.

The *E. coli* DH10B derivative strains were maintained on LB agar plates and cultured overnight at 37 °C. Specific antibiotics were used at the needed concentrations, and 80 µg/ml 5-bromo-4-chloro-3-indolyl-beta-D-galactopyranoside (X-gal) and 50 µM isopropyl-beta-D-1-thiogalactopyranoside (IPTG) were supplied in the media for the selection of strains carrying the pGEM-T Easy plasmid (Promega).

### 2.3. Design of Primers for qPCR

Using the 16S rRNA sequence from *P. putida* U (JN695040), through a BLAST search against GenBank (NCBI, https://blast.ncbi.nlm.nih.gov/Blast.cgi; last accessed 17 July 2023), different strains with the highest percentage identity were identified. Among the strains with the highest 16S rDNA identity to *P. putida* U, specific strains were selected to identify the putative highest identity for previously published genes from *P. putida* U. The best matches in the comparison were identified in the genomes from *P. putida* G7 (CP096581), *P. putida* S13.1.2 (CP010979), *P. putida* strain B1 (CP022560), *P. putida* KF715 (AP015029), *P. putida* strain GMI12077 (CP114035), *P. putida* B21-029 (CP087183), *P. putida* strain ATCC12633 (CP101910), and *P. putida* NBRC 14164 (AP013070). Once the genome with the highest identity was selected, it was used for qPCR primer design. The alignment of selected sequences was carried out using Bioedit software version 7.2.6.1 (Carlsbad, CA, USA) with native ClustalW (version 1.4, Heidelberg, Germany) [35], aligning the qPCR-amplified sequences to the *P. putida* G7 target genes.

The phylogenetic trees were constructed using the Molecular Evolutionary Genetics Analysis (MEGA) v.11.0.3 software (https://www.megasoftware.net/; Tokyo, Japan) [36,37]. Alignments of the coding genes were performed with Clustal Omega (default parameters) [38]. Phylogenetic trees were constructed using the Neighbor-Joining method [39] with the Kimura-2 parameter model [40] and the Maximum Likelihood method [41]. Bootstrap consensus trees [42] were inferred from 1000 replicates. Considering the close identity of most of the protein sequences, the use of the whole coding gene sequences was preferred. Gaps between sequences were pairwise deleted.

### 2.4. Gene Induction and RNA Extraction

To induce specific genes, *P. putida* U was cultured in the liquid minimal medium as previously described, containing as carbon sources citrate, putrescine, cadaverine, GABA, and DAVA at a final concentration of 10 mM.

For the isolation of total RNA, 10 mL of the medium was collected at an absorbance of 0.6 (corresponding to the start of the logarithmic phase of growth). Cell pellets were immediately frozen at −80 °C until the processing of the samples. The RNA was extracted using the Roche High Pure RNA Isolation Kit, and DNase treatment was performed using RNase-free Invitrogen Turbo DNase to ensure genomic DNA removal. To verify genomic DNA cleanliness, PCR checks were conducted to ensure the quality of the isolated RNA.

cDNA synthesis was performed from 5 µg of total RNA using the Thermo Scientific Maxima H Minus First Strand cDNA Synthesis Kit with random primers. The resulting cDNA was quantified using a Nanodrop 2000 (Thermo Scientific, Wilmington, DE, USA).

### 2.5. Standardizing qPCR

Analysis of the genes involved in putrescine and cadaverine degradation was conducted through relativization of the expression levels from the samples obtained as described from cultures induced by putrescine, cadaverine, or GABA concerning their expression using citrate. qPCR was performed using the Step One system (Applied Biosystems, Foster City, CA) with a final reaction volume of 20 μL [43,44]. The reaction mixture comprised 10 μL of Power SYBR Green PCR Master Mix (Applied Biosystems; Foster City, CA, U.S.A), 1 μL of 5 μmol/L Forward Primer, 1 μLof 5 μmol/L Reverse Primer (Table 1), 1 μL of 50 ng/μL cDNA, and water up the final volume of 20 μL.

Reference housekeeping genes were selected based on previous studies in other *Pseudomonas* species [45,46], and their Ct values were verified under experimental conditions. The chosen housekeeping genes were *rpoD* (σ70 transcriptional factor) and *rpoN* (σ54 transcriptional factor).

Each pair of primers designed was checked for its efficiency to ensure it fell between 90% and 110% (Table 1). Additionally, the amplicon size was verified using agarose gel electrophoresis and through Sanger sequencing (Figure 4 and Supplementary Figure S1).

Ct values of each of these genes were analyzed on each condition. Subsequently, the ΔCt from the combination of housekeeping genes was calculated on each condition to assess deviations in their values. Genes with the most stable Ct values and minimal standard deviation in their Ct increments among different conditions were selected. The analysis was performed using GraphPad Prism v. 6 software (GraphPad Software, Inc, Boston, MA, USA).

The specific primer sequences, annealing temperatures, and amplification efficiencies used in this work are provided in Table 1.

The qPCR quantification performed was relative to the expression of these genes in the presence of succinate. The formula 2^(−(ΔCt target gene −ΔCt reference gene)) was applied for relative quantification [47]. Four biological replicates were conducted to enable statistical analysis. The gene expression value in the presence of citrate was considered as the reference value (1) for normalization. GraphPad Prism 6 software (San Diego, CA, USA) was used to determine the significance of the differences observed.

### 2.6. Validation of qPCR Amplicons

The qPCR product was treated following the protocol described by Sambrook and Russel (2001). The DNA fragment was purified from an agarose gel (2.5%, *w*/*v*) using the QIAGEN GmbH purification kit. The amplified fragment was then cloned into the pGEM-T easy plasmid (Promega) for the cloning of qPCR amplicons from *P. putida* U cDNA transcripts.

Transformation of chemically competent cells from the *E. coli* DH10B strain was carried out using the RbCl method [48]. Plasmid extraction from the *E. coli* DH10 B-derived strains was performed using the QIAGEN GmbH miniprep (Antwerp, Belgium) extraction kit. Plasmid concentration and purity were determined using a Nanodrop model 6345 spectrophotometer from Thermo Fisher.

Finally, selected plasmids were sequenced using universal m13 primers flanking the multiple cloning sites from pGEM-T easy (Promega). Sanger sequencing of the different plasmids was performed by Secugen S.L. (Madrid, Spain). Sequences from the amplicons determined in this work (Figure S1) from *P. putida* U genomic DNA have been deposited in GenBank under accession numbers *spuC1* (OR435851), *spuC2* (OR435852), *gabT1* (OR435853), *gabT2* (OR435854), *rpoD* (OR435855), *rpoN* (OR435856), and *recA* (OR435857).

## 3. Results

### 3.1. Growth of P. putida U Using Putrescine, Cadaverine, GABA, DAVA, or Citrate as Carbon Sources

**Figure 2:**
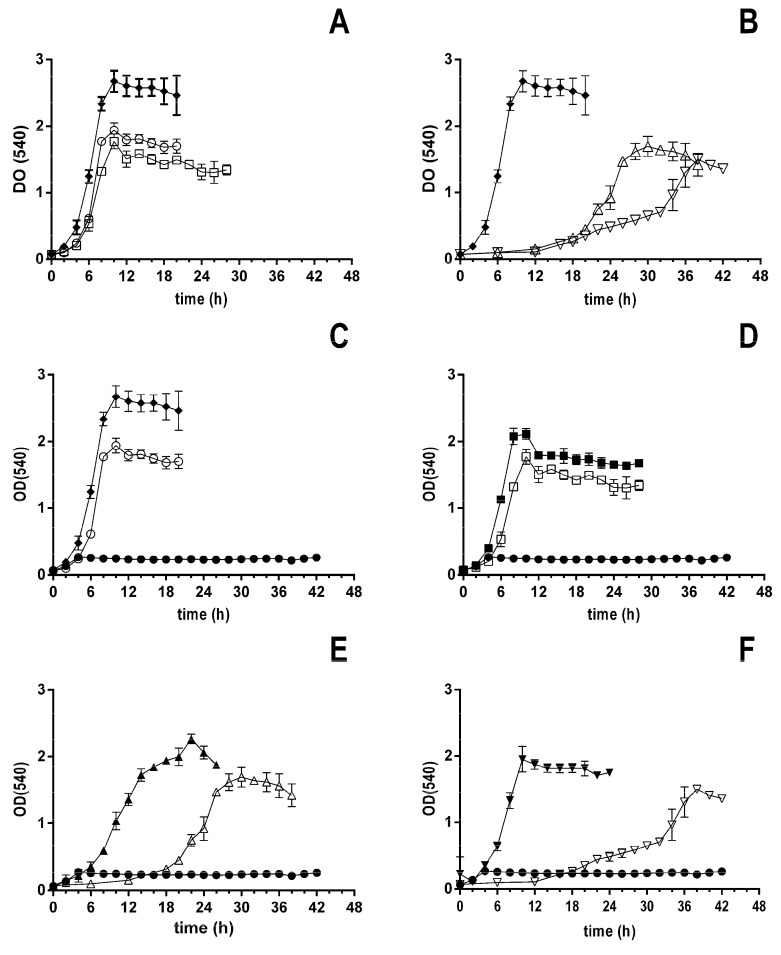
Growth of *P. putida* U determined as the increase in absorbance at 540 nm, in MM supplied with (**A**) citrate (♦), putrescine (○), and GABA (□) 10 mM; (**B**) citrate (♦), cadaverine (Δ), and DAVA (▽) 10 mM; (**C**) citrate (♦), putrescine (○) 10 mM, and putrescine (●) 1 mM; (**D**) putrescine 1 mM (♦) and GABA 10 mM with (■) and without (□) 1 mM of putrescine; (**E**) putrescine 1 mM (♦) and cadaverine 10 mM with (▲) and without (Δ) 1 mM of putrescine; (**F**) putrescine 1 mM (♦) and DAVA 10 mM with (▼) and without (▽) 1 mM of putrescine. Means and standard deviations (*n* = 3) are indicated in the growth curves. The maximum absorbance achieved for the different carbon sources is shown in Supplementary Figure S2.

The ability of the *Pseudomonas putida* strain U to metabolize polyamines and their metabolic intermediates was studied. For this purpose, *P. putida* was cultured in MM supplemented with different carbon sources (citrate, putrescine, GABA, cadaverine, and DAVA, 10 mM). The different carbon sources were used to verify the ability of *P. putida* U to use putrescine, cadaverine, and their degradation intermediates GABA and DAVA to support its growth (Figure 2). Moreover, this would allow us to start checking the degradative pathway(s) from *P. putida* U.

It was observed that *P. putida* U wild type was able to grow using all provided carbon sources (Figure 2A,B). The most prominent differences were observed in the duration of the lag phase when using different compounds. Thus, when *P. putida* U used putrescine or GABA, entry into the logarithmic phase of growth began at 8 hours, while with cadaverine or DAVA, growth was delayed at 20 hours. The latency phase was similar when cadaverine or DAVA were used, as it is with putrescine and GABA.

Once these results were verified, a second growth was performed, where all the previous growth conditions were repeated, though 1 mM putrescine was added, and control with only 1 mM putrescine was used to find out what the maximum absorbance of *P. putida* U growth would be (Figure 2C–F). The rationale of this addition was to check the possibility that 1mM putrescine could induce some genes that could also contribute to more efficient degradation of cadaverine and the other compounds. It was observed that both DAVA and cadaverine advanced their times when 1 mM putrescine was added to the medium. It should be noted that in similar experiments, the addition of 1mM cadaverine did not affect the profile of the growth curves. This finding suggested that the metabolism of cadaverine and putrescine could have some differences between them. Thus, as a first approach, the expression of the genes related to the deamination of polyamines and their putative derivatives (*spuC* and *gabT*) was proposed since these deamination processes had been proposed as the starting of polyamine, or GABA and DAVA, catabolism.

### 3.2. Search for Pseudomonas Putida U Homologous Genomes

To verify whether this improved growth is due to increased expression of the *spuC* and *gabT* genes, qPCR oligonucleotide primers were designed. However, considering that the genome of *P. putida* U was not available, these oligonucleotides had to be designed using a sequenced genome showing the highest identity to known sequences from *P. putida* U.

The first approach was to search for organisms with sequenced genomes that showed high identity with the 16S rRNA of *P. putida* U (Supplementary Table S1). Thus, different *P. putida* strains were selected, and alignment of *P. putida* U published sequences within each available allowed the selection of which of these *P. putida* strains showed the highest identity with *P. putida* U (Supplementary Table S2). After all the comparisons were made, the genome of *P. putida* G7 was selected because it showed the highest identity with all the known sequences determined in *P. putida* U.

### 3.3. Identification, Optimization, and Testing of qPCR Primers for SpuCs, GabTs, and Housekeeping Genes in P. putida U

**Figure 3:**
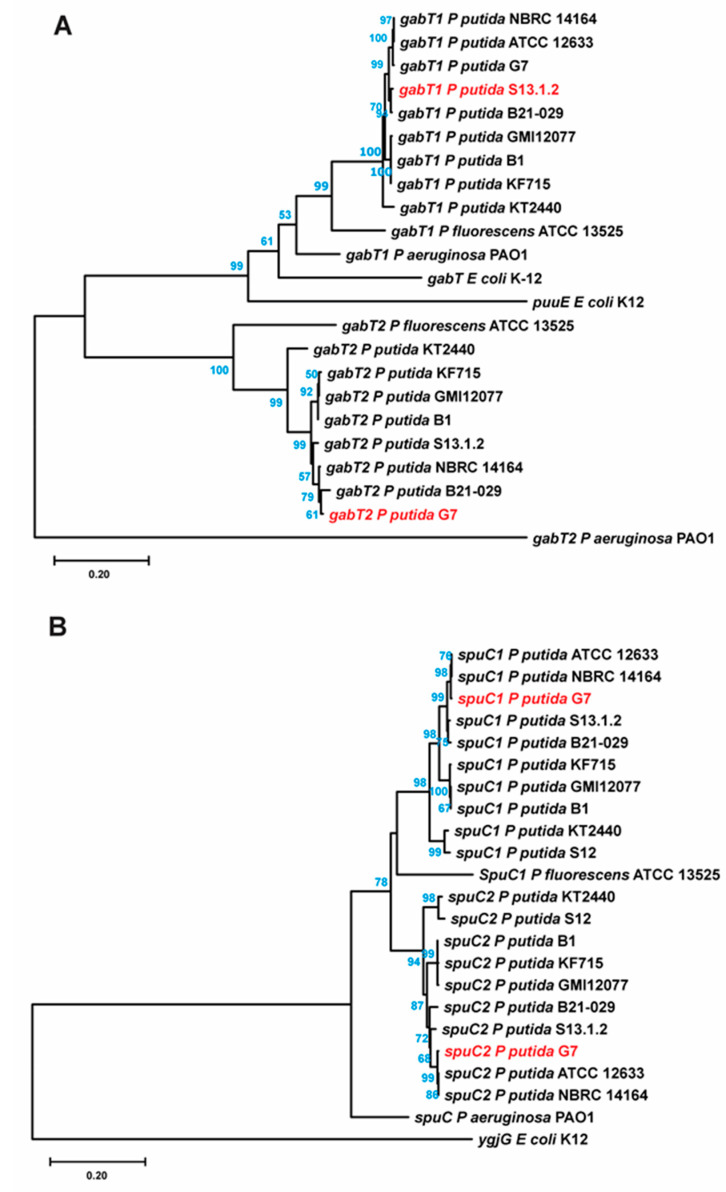
Neighbor-joining phylogenetic tree (1000 bootstrap replications) constructed using the DNA coding sequences from documented GabT proteins, as well as those found in genomes potentially close to *P. putida* U (**A**), and SpuC published in other microbiological systems and also identified in the genomes used as homologous to *P. putida* U (**B**). The tree scale (0.02) represents evolutionary distances in units of base substitutions per site as computed by the Kimura-2 parameter method. Bootstrap values of more than 50 are shown. Gaps were managed as easily paired deleted during tree construction. Trees constructed using the Maximum Likelihood method with similar bootstrapping of 1000 replicates showed a similar topology (Supplementary Figure S3). Paralog genes from *P. putida* strain G7 are indicated in red.

Once the genome with the highest identity for designing qPCR primers for *P. putida* U was selected (*P. putida* G7 genome, CP096581), the *spuC* and *gabT* genes described in *P. putida* KT2440 and S12 and in *P. aeruginosa* were searched and identified in the *P. putida* G7 genome. Thus, two putative paralogs to *spuC* (named here as *spuC1* and *spuC2*) and two possible *gabTs* (indicated as *gabT1* and *gabT2*) were identified in *P. putida* G7 (Figure 3). Using the sequences from the G7 strain, qPCR primers were designed for each of them.

**Figure 4:**
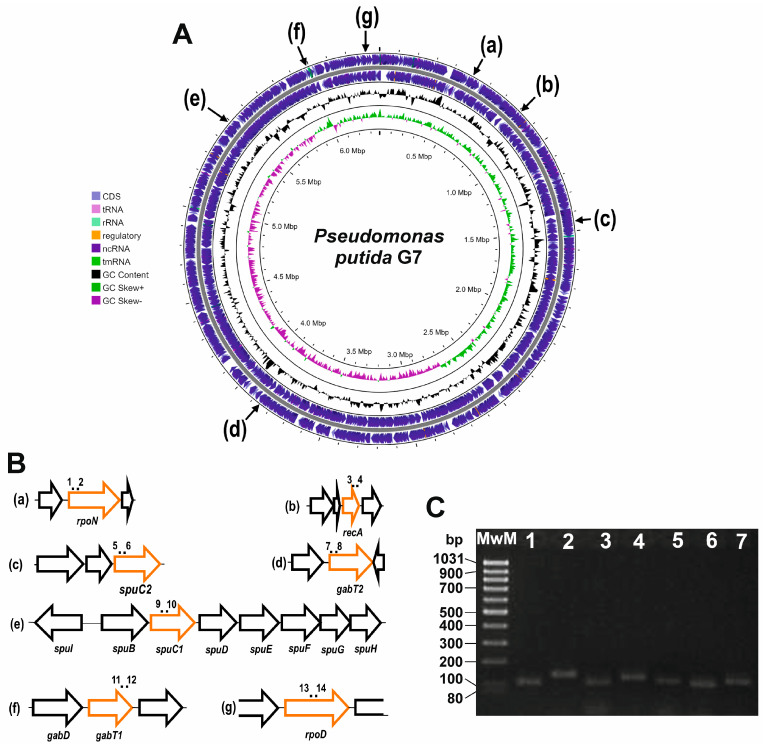
Relative position in the *P. putida* G7 genome of the genes used in the qPCR analysis. From a–g are the positions of the B-pannel genes in the *P. putida* G7 genome (**A**), and relative position of the optimal primers used for these studies (**B**). In (**C**), amplicons obtained through the qPCR were identified based on their mobility and size determined in an agarose gel (2.5%), and later, their sequence was determined through Sanger sequencing (Supplementary Figure S1). In the gel, MwM, molecular weight markers; 1. *recA* amplicon; 2. *rpoD* amplicon; 3. *rpoN* amplicon; 4. *spuC1* amplicon; 5. *spuC2* amplicon; 6. *gabT1* amplicon; 7. *gabT2* amplicon.

These putative *spuCs* and *gabTs* were used to design several pairs of primers for each studied gene in *P. putida* U. These pairs were based on different parts of the corresponding genes to then optimize and check the most optimal versions to be used in qPCR studies using the nucleic acids from *P. putida* U (Table 1 and Figure 4).

According to the phylogenetic trees performed using the *gabTs* genes (Figure 3A), as expected from the sequence conservation observed in the alignments, two different clades could be observed, one corresponding to *gabT1* sequences and the other to *gabT2*. It is noticeable at two different points: (i) *gabT1* from *E. coli* K12 showed a lower phylogenetic relationship with the sequences from pseudomonads, although clustering together, and in the same clade containing *puuE* (the corresponding gene to *gabT2* in the other bacteria) also from *E. coli*, suggesting a conserved relationship between these two paralogs; (ii) *gabT2* from *P. aeruginosa* PAO1 outgroups from both clades, suggesting a “relaxed” maintaining of the sequence concerning both gene groups and, putatively, the most dissimilar gene participating on these mechanisms.

In Figure 3B, a similar division into two different clades can be observed, showing that *spuC1* genes maintain a coherent evolutive distribution and that *spuC2* also maintains conservation between them. Thus, this suggests, due to the proximity of the two clades, that both genes arose from duplication and are maintained in the strains, at least in *P. putida*. However, *ygjG* from *E. coli* shows a clear evolutive difference from the other putrescine transaminase analyzed.

Expression analysis of genes related to the putative *spuC1* and *spuC2* from *P. putida* U was studied. The optimization of several pairs of primers for each *spuC* was performed, and one pair for each gene was selected based on their optimal performance (Figure 4). Furthermore, amplicons obtained using these primers were sequenced and aligned with the corresponding *spuCs* from the *P. putida* G7 genome (Supplementary Figure S1A,B).

A similar procedure was applied to the putative *gabT1* and *gabT2*. A specific pair of oligonucleotides was chosen for each *gabT* (Table 1). Sequencing of the amplicons obtained from *P. putida* U *gabTs* showed high identity to the *gabTs* in *P. putida* G7 (Supplementary Figure S1C,D).

Housekeeping gene selection was performed based on bibliographic references in other *Pseudomonas* species, with the *rpoN* (σ^54^ factor), *rpoD* (σ^70^ factor), and *recA* (recombinase A) genes identified as the most suitable [45,46].

For these housekeeping genes, several pairs of primers were designed based on the *P. putida* G7 sequence. Once these oligonucleotide pairs were tested, a pair was selected for each housekeeping gene (Table 1). The corresponding qPCR amplicons were analyzed by sequencing, showing high homology with the housekeeping genes identified in *P. putida* G7 (Supplementary Figure S1). After the selection of the oligonucleotide pairs, these were used to study the variation presented by each possible housekeeping gene under all study conditions, ultimately selecting only two housekeeping genes (*rpoN* and *rpoD*) based on their high stability in the study conditions, in agreement with different studies from other laboratories [45,46].

### 3.4. Expression Study of SpuCs and GabTs in Pseudomonas Putida U

The analysis was based on the study of the expression of each of the genes *spuC1*, *spuC2*, *gabT1*, and *gabT2* in media with 10 mM citrate as the control, 10 mM putrescine, 10 mM cadaverine, 10 mM GABA, and 10 mM DAVA. All conditions were referenced to the control condition (10 mM citrate).

**Figure 5:**
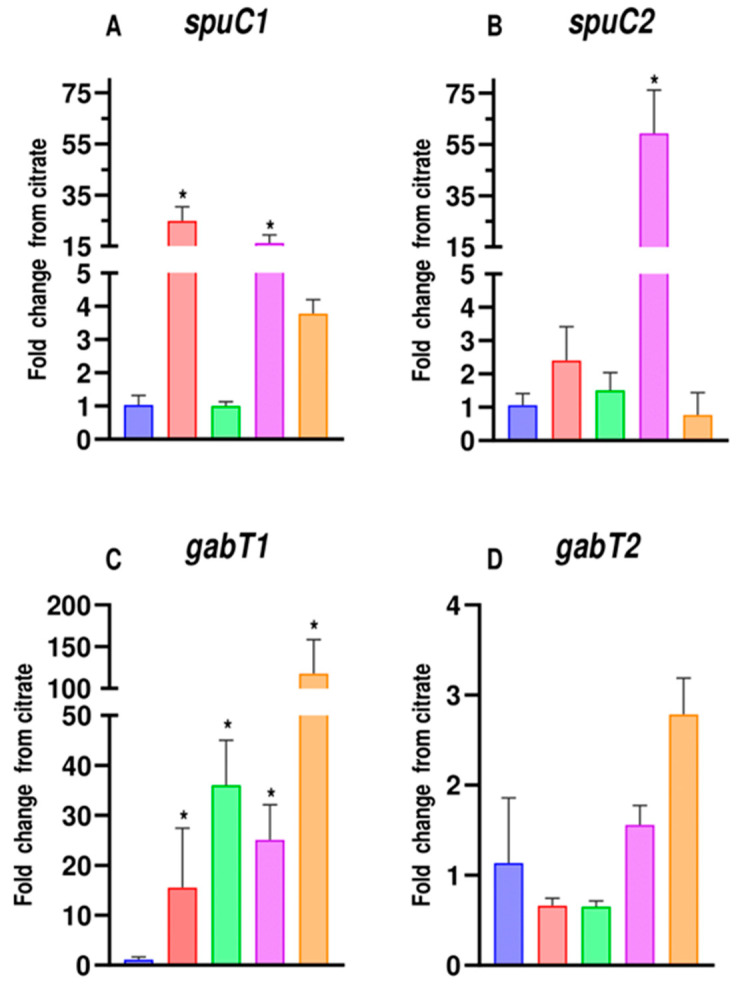
Fold change of expression of *spuC1* (**A**), *spuC2* (**B**), *gabT1* (**C**), and *gabT2* (**D**) genes analyzed when *P. putida* U was cultured in citrate (blue), putrescine (red), GABA (green), cadaverine (purple), or DAVA (orange) at 10 mM as sole carbon and energy source. Values are means ± standard deviation (SD) (*n* = 4). Statistical significance was determined with ANOVA for citrate; * is *p* < 0.05.

After normalization of the results obtained from the qPCR concerning the expression of the selected housekeeping genes, upregulation of both *spuC1* and *spuC2* was observed when the bacteria were cultured using cadaverine or DAVA. However, only *spuC1* was upregulated in those media containing putrescine or GABA. Profile of regulation of these genes in the presence of GABA and DAVA makes sense, considering that both compounds are catabolic intermediates in the degradative pathways of the corresponding polyamines. Moreover, *spuC2* was only induced with cadaverine and not with putrescine (Figure 5A,B).

Furthermore, the expression of *gabT1* appears to be increased compared to citrate in putrescine, GABA, cadaverine, and DAVA. In contrast, *gabT2* seems to be unaffected in expression relative to citrate with putrescine, GABA, cadaverine, and DAVA (Figure 5C,D).

Overall, the study identifies two *spuC* paralogs and verifies that the promoter triggering the expression of *spuC2* responded exclusively, in the conditions tested, to the presence of cadaverine, whereas the control of *spuC1* promoter seems to be more relaxed for the presence of different polyamines. The results shed light on the expression patterns of these genes under different conditions, providing valuable insights into their functions in the degradation pathway of amines.

### 3.5. Analysis of the Putative Cluster Containing SpuC1

After the qPCR analysis and regarding the growth curves, it is evident that *spuC1* is induced by putrescine, thereby enhancing the degradation efficiency of cadaverine and DAVA, as observed in the growth curves. However, the literature indicates that the *spuC* genes are capable of producing the deamination of both putrescine and cadaverine [29,49]. Thus, one potential way for putrescine to improve the growth using DAVA as a carbon source could be through transportation. Indeed, the PotABCD transporters, as supported by the literature, are proficient in transporting various polyamines and primary amines [50].

**Figure 6:**
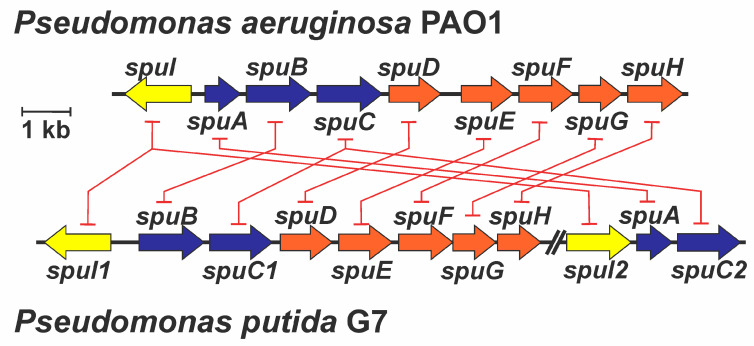
Schematic representation of the *spu* cluster of genes and in *P. aeruginosa* PAO1 and *P. putida* G7 showing the genetic rearrangements between both strains. Orange color indicates genes putatively involved in the uptake of polyamines, whereas blue color indicates genes whose products participate in polyamine catabolic processes. Yellow color (*spuI* genes) indicates genes that putatively encoded glutamine synthetase-like proteins that, although in the literature about catabolism of polyamines in *Pseudomonas* have been suggested as participants in the polyamine catabolic process, Krysenko et al. demonstrated, in *S. coelicolor*, their participation in γ-glutamylation of polyamines and other monoamines [18]. Red lines indicate the genetic rearrangements affecting genes in both bacteria, evidencing the presence of some paralog genes in *P. putida* G7. NOTE: During the processing of this article, authors have confirmed through PCR amplification using degenerate primers designed based on the genome of *P. putida* G7, the maintaining of the synteny to the corresponding genes between G7 strain genes and those present in the genome of *P. putida* U (data not shown).

For this reason, a study on the adjacent genes to *spuC1* in *P. putida* G7 was conducted, showing that the ABC-like transporter genes possibly belong to the same cluster as *spuC1*. A similar finding has been documented as happens in *P. aeruginosa* PAO1, where transport genes have been described to be under the control of the same promoter between *spuI* and *spuA*, where it expresses all *spuABCDEFGH* genes [26]. The main difference between *P. putida* G7 and *P. aeruginosa* corresponds to the synteny of the *spuA* gene since, in *P. putida* G7, this gene is located in a cluster close to the *spuC2* gene. This rearrangement suggests the possibility of a potential increase in the expression levels of both the transport system and *spuC1* in response to the presence of putrescine (Figure 6). Thus, the heightened activation of the *spuC1* cluster expression could be potentially attributed to a higher efficiency of the uptake of putrescine by the periplasmic PotF protein compared to cadaverine [51].

## 4. Discussion

*Pseudomonas putida* U is a bacterium known for its outstanding degradative ability, especially towards various amines [17]. This study demonstrated its capacity to degrade compounds such as putrescine, cadaverine, and their degradation intermediates GABA and DAVA, as described in other organisms [17,27,28,52,53].

The obtained results regarding the growth of *P. putida* U in different amines present relevant data, such as different growth times between putrescine/GABA and cadaverine/DAVA. Although previous studies suggest the same degradation pathway for putrescine and cadaverine, they do not mention differences in the efficiency of degradation for these amines and relatives to grow [29]. It has been referenced in the literature that the expression of the *spuABCDEFGH* coexpressed when putrescine is catabolically used and, consequently, forms an operonic unit, or these genes are organized as different transcriptional units under the same regulatory mechanism [27]. This suggests that, at least in different pseudomonads, both cadaverine and putrescine are metabolized by the same pathway, being assimilated at a similar rate. However, the results presented here suggest a different behavior of *P. putida* U in the metabolism of cadaverine and putrescine. Based on that, a similar growth kinetic determination was performed, although with the addition of a low concentration of putrescine. The rationale for this was the assumption that, as indicated for *P. aeruginosa*, putrescine could act as an inducer of the expression of the entire degradation pathway [27]. The growth in cadaverine and DAVA using these conditions was faster, suggesting a faster induction of the degradative pathway by putrescine or GABA than by cadaverine or DAVA.

The first putrescine and cadaverine degradative process is carried out by amino oxidoreductase or aminotransferases. In *P. aeruginosa*, the proteins PauB1-4 and SpuC have been described as responsible for these initial deaminations. In *P. putida* G7, homologs of *pauB* were found, but we focused on the *spuC* genes since two *spuC* paralog genes were found, homologous to the only one described in *P. aeruginosa* as associated with the deamination of both putrescine and cadaverine [54]. These *spuC* genes showed different expression profiles triggered by different amines in *P. putida* U. *spuC1* in *P. putida* U is more related to putrescine than cadaverine and is unrelated to GABA and DAVA, as inferred from the *E. coli* and *P. aeruginosa* pathways [17,21]. On the other hand, the fact that one *spuC* is upregulated specifically by putrescine could explain the slower growth in cadaverine mentioned earlier. However, the gene *spuC2* seems to be exclusively induced in response to cadaverine and not to putrescine. Therefore, this would be the first evidence of a *spuC1* induced by both amines and a *spuC2* exclusive to cadaverine.

The existence of two *spuC* paralogs seems to be conserved in other *P. putida* strains, where the *spu1* clusters resemble the *spuABCDEFGH* cluster of *P. aeruginosa* [27], with the transporter genes linked, suggesting a co-transcription of the *spuC1* gene of *P. putida* and the *spuDEFGH* genes involved in polyamine transport. On the other hand, the possible *spu2* cluster, when performing a homology analysis, shows us three genes: one that potentially would be *spuC2* and the other two genes potentially participating in the ɣ-glutamyl-degrading mechanism (Figure 6). This could explain why putrescine allows faster growth than cadaverine, as putrescine increases the transporters by increasing the transcript of *spuC1*, which belongs to the *spu1* cluster (Figure 5A). Regarding enzymatic activity, no differences were found between cadaverine and putrescine at some points of the pathway, as suggested in the literature [54]. Therefore, this growth delay is mainly due to gene induction and possibly less efficient transport of these transporters, where one of their periplasmic polyamine binding proteins, SpuF, could show a higher affinity for putrescine than cadaverine [51].

Furthermore, a study of *gabT* genes was conducted, as they are responsible for the first degradation step of the GABA and DAVA intermediates [17,24]. Through alignments, two *gabTs* paralogs were found, resembling what is indicated in the literature for *E. coli* [24,55]. The expression results in cadaverine, putrescine, DAVA, and GABA indicate that although the *gabT1* gene is upregulated, *gabT2* is not. Therefore, these results suggest that *gabT1* could be an inducible gene, while *gabT2* seems to show a constitutive expression, as described in *E. coli* [25]. However, it is still uncertain whether putrescine and cadaverine induce by themselves the expression of *gabT* genes or if the true inducer of some of the genes could be a catabolic intermediate (GABA and/or DAVA), unlike *E. coli*, where putrescine, but not GABA, has been indicated to be the inducer of the expression of *gabT* [25].

In *P. putida* G7, two degradative gene clusters can be identified. Cluster *spu1* contains all the necessary genes for the transport, deamination, glutamylation, and aldehyde dehydrogenase required for amine degradation. However, it appears that this possible cluster is more strongly induced by putrescine than cadaverine in *P. putida* U. On the other hand, the second cluster, *spu2*, contains two genes involved in polyamine glutamylation and a gene encoding an aminotransferase (*spuC2*) that seems to be expressed only in response to cadaverine.

Finally, this work opens a path to improving cadaverine degradation by identifying specific genes for this polyamine, allowing the study of the enzymatic activity of these genes and potentially enhancing the degradative activity of cadaverine compared to genes induced by both polyamines.

## 5. Conclusions

*P. putida* U is a bacterium able to catabolize putrescine and cadaverine, as well as their intermediates GABA and DAVA, supporting its growth, as previously described in other organisms [17,18]. The known genetic sequences of *P. putida* U exhibit high identity with those from the *P. putida* G7 genome, enabling the design of oligonucleotide primers for the study of specific genes. This, in conjunction with the maintaining of specific genes in the *P. aeruginosa* PAO1 genome, suggests that although the canonical pathways are maintained in pseudomonads, some strain-specific modifications could occur in the catabolism of different polyamines.

Two different polyamine aminotransferases involved in the metabolism of putrescine and cadaverine have been identified in *P. putida* strain U. The gene *spuC1* is expressed in the presence of both polyamines, while the gene *spuC2* is only upregulated by cadaverine. This implicates a specific gene response involvement of *spuC2* in cadaverine deamination.

In comparison to *P. aeruginosa*, where a single gene cluster responsible for the transport and catabolism of polyamines has been proposed [27], in *P. putida,* two distinct genetic clusters have been identified until now. The *spu1* cluster contains a *spuC1* gene, other genes related to γ-glutamylation, and a polyamine transport system that shows homology with the one described in *P. aeruginosa*. On the other hand, the *spu2* cluster contains two genes involved in γ-glutamylation and the *spuC2* gene, whose expression responds exclusively to the presence of cadaverine in *P. putida* U. The *spuBCDEFGH* operon from *P. putida* U is analogous to the one described in *P. aeruginosa* [26], except for the rearrangement in the genome of the *spuA* gene that is found in the *spu2* cluster in *P. putida* (Figure 6).

These genetic differences between *P. putida* and *P. aeruginosa* suggest that *P. putida* has evolved a more complex strategy for polyamine metabolism, potentially influencing its degradative capacity and adaptation to different environmental conditions. The results here suggest that the study of the genes and their functions, as well as the arrangement of metabolic functions suggesting networking participation of paralog functions in a differential way depending on the polyamine to be catabolized in *P. putida* U, may provide a deeper understanding of bacterial polyamine degradation. However, we are conscious that more studies are necessary to establish the complex interactions of these pathways in our bacterial model; the potential results could help for a better understanding of the complexity of polyamine metabolism, with an impact on the food industry and the development of infection therapies.

## Figures and Tables

**Table 1:** Oligonucleotide primers designed, and their main characteristics, used for the analysis of differential expression of some genes in *P. putida* in response to polyamines or catabolic derivatives from them. The annealing temperature used for all of them was 60 °C.

Oligonucleotide	Target Gene	Sequence (5’–3’)	Amplicon Size (bp)	Efficiency (%)	R^2^ of Efficiencies
Q-SpuC1,1-5′ PPU	*spuC1*	AAGCCGCACAAGCAGACCAT	132 pb	96.59	0.990
Q-SpuC1,1-3′ PPU		GATGTGCACGATGCCCGGAA			
Q-SpuC2,1-5′ PPU	*spuC2*	CCAAGGGCGTGTACCTGTGG	122 pb	95.20	0.986
Q-SpuC2,1-3′ PPU		CATCTGCTGGCTGGCAACCT			
F1.gabT1.PPU	*gabT1*	GCTGTTGGCCAGGTTGTTGC	144 pb	98.82	0.998
R1.gabT1.PPU		CAGTGCGTCTTCGGCAGTCA			
F1.gabT2.PPU	*gabT2*	CGCCATCGTTCACCCGATCA	120 pb	93.92	0.992
R1.gabT2.PPU		TTGCAGTGGCCCAGGTTGAG			
F1.rpoD.PPU	*rpoD*	GCGAACCTGCGTCTGGTGAT	138 pb	96.14	0.997
R1.rpoD.PPU		GAACTTGTAGCCGCGACGGT			
F1.rpoN.PPU	*rpoN*	TCGAACCCGATGCTCGAACG	109 pb	101.01	0.994
R1.rpoN.PPU		AGCTGTTGTCCTGGGCTTCG			
F1.recA.PPU	*recA*	GGCGAACAGGCCCTGGAAAT	107 pb	99.42	0.988
R1.recA.PPU		CCTTCGATCTCGGCCTTGGG			

## Data Availability

All relevant data are included in the manuscript and Supplementary Files. New sequences have been sent to GenBank (NCBI, Bethesda, USA).

## Supplementary Materials

The following supporting information can be downloaded at https://www.mdpi.com/article/10.3390/genes14101897/s1. Figure S1: Alignment of the DNA sequences corresponding to the amplicons obtained in *P. putida* U by qPCR using the primers for *spuC1*, *spuC2*, *gabT1*, and *gabT2* with the corresponding sequences from *P. putida* G7 genome (CP096581); Figure S2: Maximal growth reached by *P. putida* U, determined as absorbance at 540 nm, when growing in MM supplied with different polyamines, or derivatives, as sole carbon source. Figure S3: Phylogenetic analysis of the selected *gabT1* and *gabT2* gene sequences (A) and *spuC* genes (B) using the Maximum Likelihood method and Kimura-2 parameter model, using a bootstrapping of 1000. Bootstrap values of more than 50 are shown; Table S1: Identity percentages obtained from the alignment of 16S rRNA sequence from *P. putida* U against other *P. putida* strain sequences from GenBank (NCBI) showing that the higher identity was reached with *P. putida* G7; Table S2: Identities obtained through the alignment of the *P. putida* U DNA sequences present in the GenBank database against their orthologs from *P. putida* G7 and *P. putida* S13.1.2 genomes. Refs [56,57,58,59,60,61] have been cited in this file.
