# Supplementary material for: Specific Gene Expression in Pseudomonas Putida U Shows New Alternatives for Cadaverine and Putrescine Catabolism
Source: Genes (Basel). 2023 Sep 30;14(10):1897. doi: 10.3390/genes14101897 (PMC10606097; doi:10.3390/genes14101897)
Supplement: Supplementary file 1 [file genes-14-01897-s001.zip › genes-2586409-supplementary.pdf]

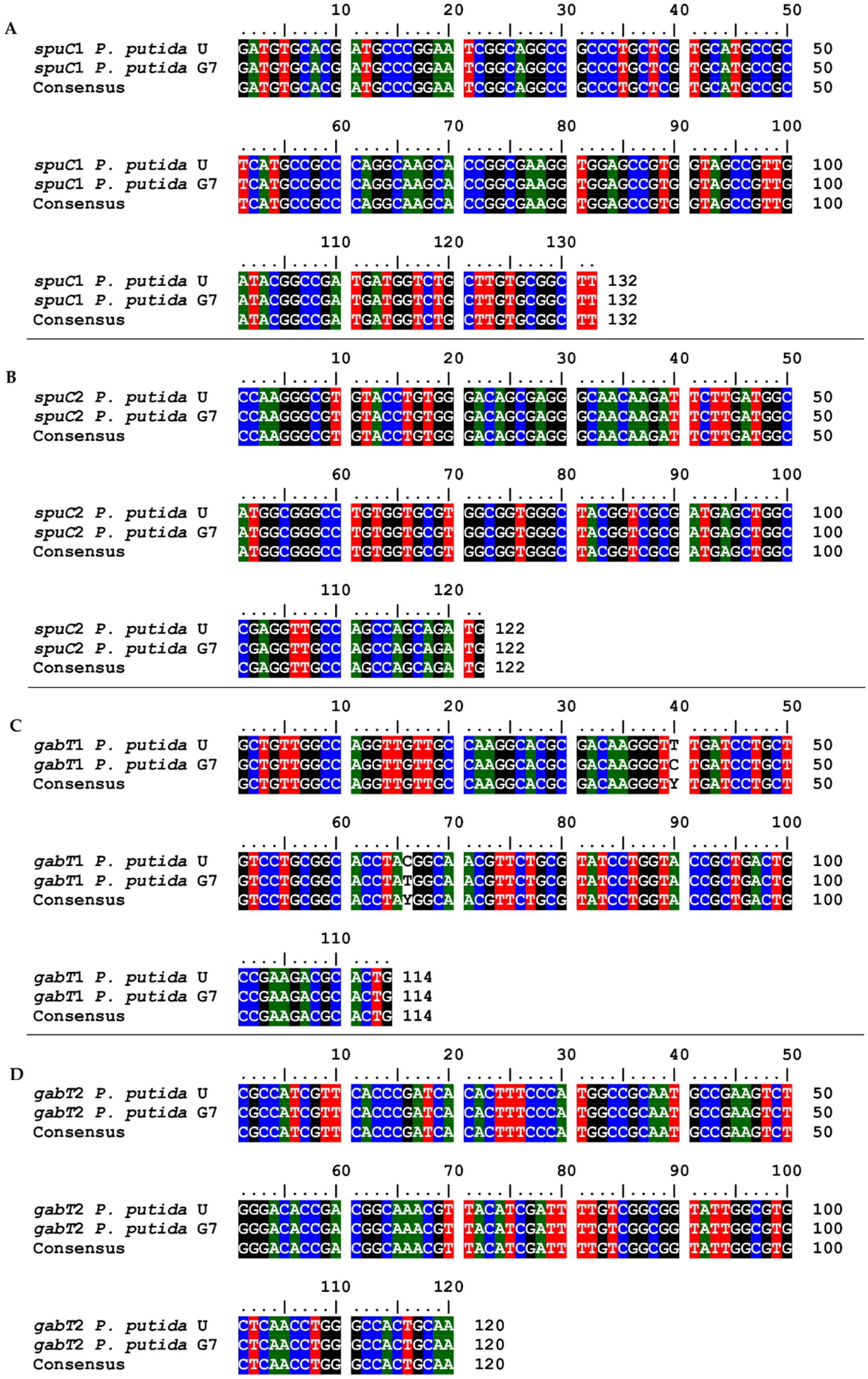

Figure S1. Alignment of the DNA sequences corresponding to the amplicons obtained in *P. putida* U by qPCR using the primers for *spuC1*, *spuC2*, *gabT1*, and *gabT2* with the corresponding sequences from P. putida G7 genome (CP096581). *spuC1* (locus tag M0766_26090 in *P. putida* G7, OR435851 sequence ID in GenBank for *P. putida* U sequence), *spuC*2 (G7 strain, M0766_07040, U strain OR435852), *gabT*1 (G7 strain M0766_28610, U strain OR435853), and *gabT*2 (G7 strain, M0766_18295, U strain OR435854).

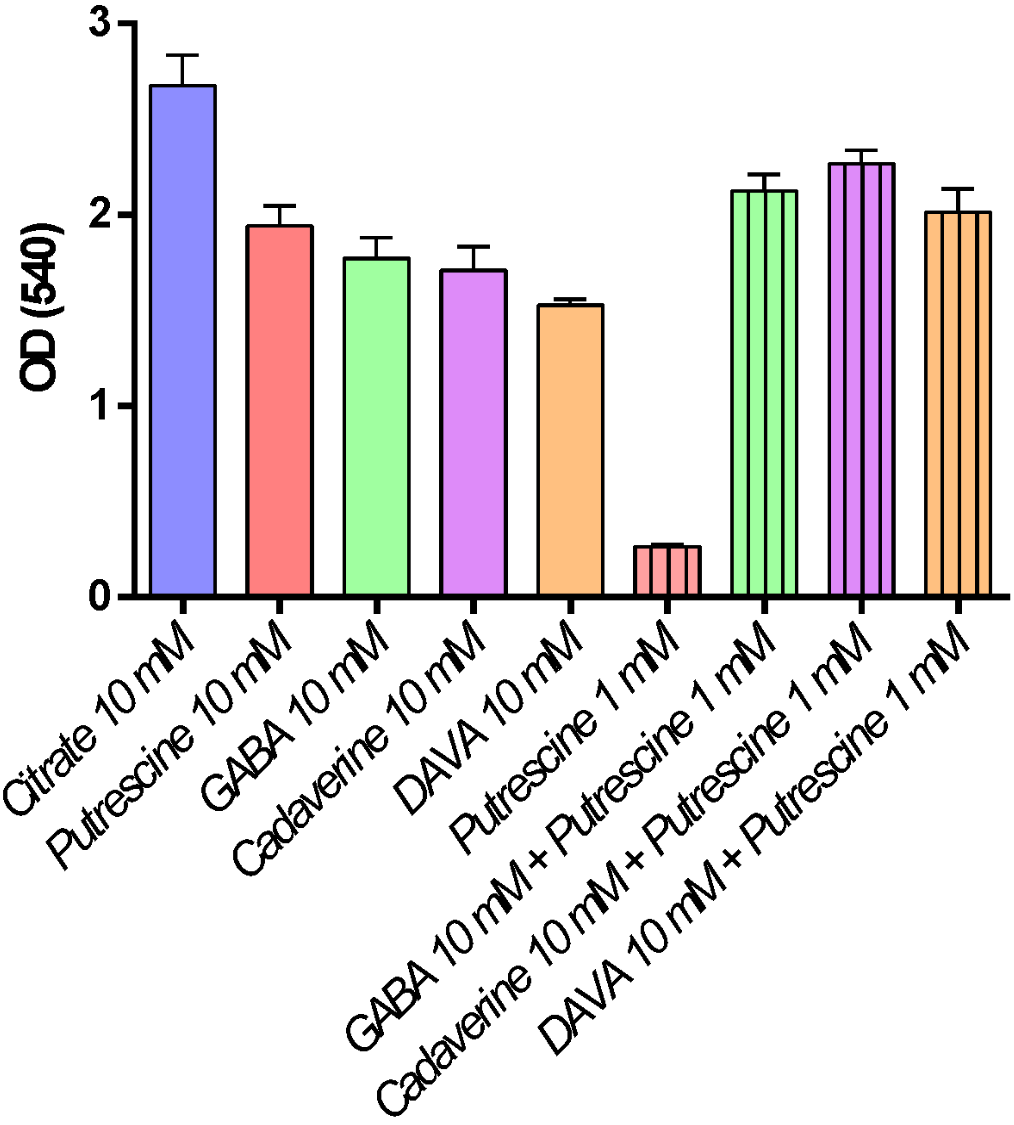

Figure S2. Maximal growth reached by *P. putida* U, determined as absorbance at 540 nm, when growing in MM supplied with different polyamines, or derivatives, as sole carbon source.

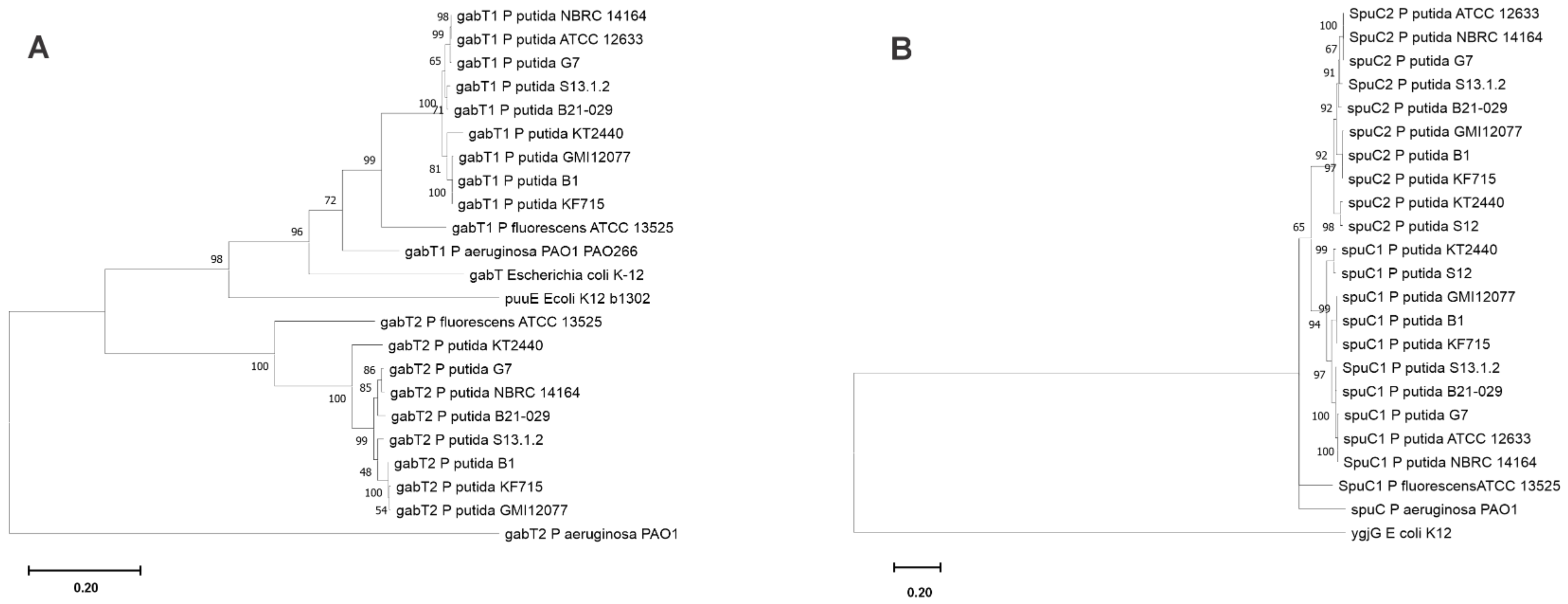

Figure S3. Phylogenetic analysis of the selected *gabT1* and *gabT2* gene sequences (A) and *spuC* genes (B) using the Maximum likelihood method and Kimura-2 parameter model, using a bootstrapping of 1000. Bootstrap values of more than 50 are shown.

Table S1. Identity percentages obtained from the alignment of 16S rDNA sequence from *P. putida* U against other *P. putida* strain sequences from GenBank (NCBI). *P. putida* S13.1.2 (CP010979), *P. putida* G7 (CP096581), *P. putida* ZH9 (MN094380), *P. putida* SAs-14 (JQ782896), *P. putida* JCM13061 (LC507958), *P. putida* ZJ15-14 (OM533711), *P. putida* NBRC14164 (NR_113651), *P. putida* B1 (CP022560), *P. putida* 4A1 (MH379785) and *P. putida* KF715 (AP015029).

Strain	Per. Identity	Strain	Per. Identity
P. putida S13.1.2	100	*P. putida* G7	100
P. putida ZH9	100	*P. putida* SAs-14	100
P. putida JCM13061	100	*P. putida* ZJ15-14	99
P. putida NBRC14164	99	*P. putida* B1	99
P. putida 4A1	99	*P. putida* KF715	99

Table S2. Identities obtained between the alignments of the *P. putida* U DNA sequences present in the GenBank database against their orthologs from *P. putida* G7 and *P. putida* S13.1.2 genomes.

Accesion (and size)	G7	S13.1.2	Reference
AY929299 (6539 bp)	3512/3605(97%)	4348/4581(95%)	[1]
EF406121 (19360 bp)	19314/19358(99%)	9124/9491(96%) 5262/5429(97%) 3458/3654(95%)	[2]
EF406120 (17832 pb)	9696/9830(99%) 5644/5817(97%) 1015/1040(98%)	9226/9805(94%) 5178/5725(90%) 1015/1040(98%)	
MF325142 (7866 pb)	7817/7867(99%)	5803/6019(96%) 1765/1835(96%)	[3]
MF325143 (4800 pb)	4795/4803(99%)	4010/4147(97%)	
MF325144 (4200 pb)	4194/4200(99%)	4083/4200(97%)	
MF325145 (1332 pb)	1323/1331(99%)	1273/1332(96%)	
MF325146 (5755 pb)	5633/5766(98%)	5552/5780(96%)	
MF325147 (4672 pb)	4654/4672(99%)	4357/4540(96%)	
JN695045 (1186 pb)	1183/1186(99%)	1168/1186(98%)	[4]
**JN695040.1 (1460)**	**1460/1460(100%)**	**1460/1460(100%)**	
AF029714 (23521 pb)	20208/20296(99%)	9725/10257(95%) 5110/5318(96%) 1477/1656(89%) 1308/1543(85%)	[5]
AF150671 (1440 pb)	1420/1438(99%)	1413/1440(98%)	[6]
AF150672 (1215 pb)	1212/1215(99%)	1174/1215(97%)	
AF150669 (1920 pb)	1912/1920(99%)	1852/1920(96%)	
FJ904934 (25132 pb)	15727/15854(99%) 9126/9175(99%)	13551/14138(96%) 4303/4482(96%) 3053/3236(94%) 2952/3098(95%)	[7]
AY168853 (1232 pb)	1229/1232(99%)	1201/1232(97%)	[8]
AY168852 (5861 pb)	5813/5850(99%)	5590/5856(95%)	
AY168854 (1191 pb)	1190/1191(99%)	1107/1195(93%)	
AY168855 (4373 pb)	4344/4373(99%)	4241/4373(97%)	
AF290948 (4207 pb)	4158/4210(99%)	2041/2120(96%) 1924/2008(96%)	[9]
AF290949 (3963 pb)	3947/3963(99%)	3731/3849(97%)	
AF290950 (5686 pb)	5661/5687(99%)	5293/5686(93%)	

Supplementary bibliographic references

1. Arias-Barrau, E.; Sandoval, A.; Naharro, G.; Olivera, E.R.; Luengo, J.M. A two-component hydroxylase involved in the assimilation of 3-hydroxyphenyl acetate in *Pseudomonas putida*. *J Biol Chem* **2005**, *280*, 26435-26447, doi:10.1074/jbc.M501988200.
2. Arias, S.; Olivera, E.R.; Arcos, M.; Naharro, G.; Luengo, J.M. Genetic analyses and molecular characterization of the pathways involved in the conversion of 2-phenylethylamine and 2-phenylethanol into phenylacetic acid in *Pseudomonas putida* U. *Environ Microbiol* **2008**, *10*, 413-432, doi:10.1111/j.1462-2920.2007.01464.x.
3. de la Torre, M.; Gómez-Botrán, J.L.; Olivera, E.R.; Bermejo, F.; Rodríguez-Morán, J.; Luengo, J.M. Histamine catabolism in *Pseudomonas putida* U: identification of the genes, catabolic enzymes and regulators. *Environ Microbiol* **2018**, *20*, 1828-1841, doi:10.1111/1462-2920.14118.
4. Merino, E.; Barrientos, A.; Rodriguez, J.; Naharro, G.; Luengo, J.M.; Olivera, E.R. Isolation of cholesterol- and deoxycholate-degrading bacteria from soil samples: evidence of a common pathway. *Applied Microbiology and Biotechnology* **2013**, *97*, 891-904, doi:10.1007/s00253-012-3966-7.
5. Olivera, E.R.; Minambres, B.; Garcia, B.; Muniz, C.; Moreno, M.A.; Ferrandez, A.; Diaz, E.; Garcia, J.L.; Luengo, J.M. Molecular characterization of the phenylacetic acid catabolic pathway in *Pseudomonas putida* U: The phenylacetyl-CoA catabolon. *Proceedings of the National Academy of Sciences of the United States of America* **1998**, *95*, 6419-6424, doi:10.1073/pnas.95.11.6419.
6. Garcia, B.; Olivera, E.R.; Minambres, B.; Fernandez-Valverde, M.; Canedo, L.M.; Prieto, M.A.; Garcia, J.L.; Martinez, M.; Luengo, J.M. Novel biodegradable aromatic plastics from a bacterial source: Genetic and biochemical studies on a route of the phenylacetyl-CoA catabolon. *Journal of Biological Chemistry* **1999**, *274*, 29228-29241, doi:10.1074/jbc.274.41.29228.
7. Arcos, M.; Olivera, E.R.; Arias, S.; Naharro, G.; Luengo, J.M. The 3,4-dihydroxyphenylacetic acid catabolon, a catabolic unit for degradation of biogenic amines tyramine and dopamine in *Pseudomonas putida* U. *Environmental Microbiology* **2010**, no-no, doi:10.1111/j.1462-2920.2010.02233.x.
8. Arias-Barrau, E.; Olivera, E.R.; Luengo, J.M.; Fernandez, C.; Galan, B.; Garcia, J.L.; Diaz, E.; Minambres, B. The homogentisate pathway: A central catabolic pathway involved in the degradation of L-phenylalanine, L-tyrosine, and 3-hydroxyphenylacetate in *Pseudomonas putida*. *Journal of Bacteriology* **2004**, *186*, 5062-5077, doi:10.1128/jb.186.15.5062-5077.2004.
9. Olivera, E.R.; Carnicero, D.; Garcia, B.; Minambres, B.; Moreno, M.A.; Canedo, L.; DiRusso, C.C.; Naharro, G.; Luengo, J.M. Two different pathways are involved in the beta-oxidation of n-alkanoic and n-phenylalkanoic acids in *Pseudomonas putida* U: genetic studies and biotechnological applications. *Molecular Microbiology* **2001**, *39*, 863-874, doi:10.1046/j.1365-2958.2001.02296.x.
